# Immunogenicity and Protective Efficacy of a Recombinant Pichinde Viral-Vectored Vaccine Expressing Influenza Virus Hemagglutinin Antigen in Pigs

**DOI:** 10.3390/vaccines10091400

**Published:** 2022-08-26

**Authors:** Sushmita Kumari, Jayeshbhai Chaudhari, Qinfeng Huang, Phillip Gauger, Marcelo Nunes De Almeida, Yuying Liang, Hinh Ly, Hiep L. X. Vu

**Affiliations:** 1 Nebraska Center for Virology, University of Nebraska-Lincoln, Lincoln, NE 68583, USA; 2 School of Veterinary Medicine and Biomedical Sciences, University of Nebraska-Lincoln, Lincoln, NE 68583, USA; 3 Veterinary & Biomedical Sciences Department, College of Veterinary Medicine, University of Minnesota, Twin Cities, MN 55108, USA; 4 Veterinary Diagnostic Laboratory, College of Veterinary Medicine, Iowa State University, Ames, IA 50011, USA; 5 Department of Animals Sciences, University of Nebraska-Lincoln, Lincoln, NE 68583, USA

**Keywords:** swine influenza, vaccine, hemagglutinin, pichinde virus, arenavirus, viral vector vaccine

## Abstract

Influenza A virus of swine (IAV-S) is an economically important swine pathogen. The IAV-S hemagglutinin (HA) surface protein is the main target for vaccine development. In this study, we evaluated the feasibility of using the recombinant tri-segmented Pichinde virus (rPICV) as a viral vector to deliver HA antigen to protect pigs against IAV-S challenge. Four groups of weaned pigs (T01–T04) were included in the study. T01 was injected with PBS to serve as a non-vaccinated control. T02 was inoculated with rPICV expressing green fluorescence protein (rPICV-GFP). T03 was vaccinated with rPICV expressing the HA antigen of the IAV-S H3N2 strain (rPICV-H3). T04 was vaccinated with the recombinant HA protein antigen of the same H3N2 strain. Pigs were vaccinated twice at day 0 and day 21 and challenged at day 43 by intra-tracheal inoculation with the homologous H3N2 IAV-S strain. After vaccination, all pigs in T03 and T04 groups were seroconverted and exhibited high titers of plasma neutralizing antibodies. After challenge, high levels of IAV-S RNA were detected in the nasal swabs and bronchioalveolar lavage fluid of pigs in T01 and T02 but not in the T03 and T04 groups. Similarly, lung lesions were observed in T01 and T02, but not in the T03 and T04 groups. No significant difference in terms of protection was observed between the T03 and T04 group. Collectively, our results demonstrate that the rPICV-H3 vectored vaccine elicited protective immunity against IAV-S challenge. This study shows that rPICV is a promising viral vector for the development of vaccines against IAV-S.

## 1. Introduction

Influenza A virus of swine (IAV-S) is one of the most important respiratory pathogens of swine [1]. The virus is widespread worldwide, causing significant economic loss to swine producers [2]. Clinically, pigs infected with IAV-S often display signs of an acute respiratory disease that rapidly resolves after 7 or 10 days of infection. However, when associated with other pathogens of the porcine respiratory disease complex, IAV-S infection can often lead to severe pneumonia [3]. There are three known major subtypes of IAV-S affecting pigs: H1N1, H1N2, and H3N2. Based on phylogenetic analysis of the IAV-S hemagglutinin (HA) sequences, the H1 and H3 subtypes can be further classified into multiple clades or lineages that are genetically distant from one another [4,5,6,7]. New strains of influenza virus frequently emerge in swine herds [8]. Genetic diversity of IAV-S represents a major hurdle for developing an efficacious vaccine [9,10,11].

Different platforms have been considered and used towards the development of vaccines against IAV [12]. Polyvalent, whole-inactivated virus (WIV) [13] vaccines are commonly used for the control of IAV-S infection [14]. The WIV vaccines are found to be effective in protecting vaccinated pigs against antigenically matched IAV-S strains, but vaccine efficacy dramatically decreases when vaccinated pigs are exposed to antigenically mismatched virus strains [15,16,17]. Additionally, the use of WIV vaccines may enhance the severity of clinical outcomes if the vaccinated pigs are subsequently exposed to mismatched IAV-S strains of the same subtype [16,18], a phenomenon known as vaccine-associated enhanced respiratory disease (VAERD). Recently, a live-attenuated virus (LAV) vaccine against IAV-S was licensed for clinical application [19,20]. Experimental data demonstrate that the LAV vaccine could confer better heterologous protection than WIV vaccines [21,22,23]. However, reassortment between LAV and field IAV-S isolates has been documented, raising a real concern regarding the safety of the LAV vaccine platform [24].

HA is an important target for the development of IAV-S vaccines. Different forms of the HA antigen have been tested in pigs as subunit vaccines, including HA protein- and DNA-based vaccines as well as viral vector-based vaccines expressing HA [25,26,27,28,29,30]. Several viral vectors have been employed to deliver HA antigen in pigs that include but are not limited to replication-defective human adenovirus type 5 (Ad5) and Orf virus [31,32].

We are interested in developing Pichinde virus (PICV) as a viral vector to deliver HA antigen in pigs. PICV is an enveloped RNA virus within the *Arenaviridae* family. The virus was first isolated from rice rats (*Oryzomys albigularis*) in Colombia, South America [33]. PICV is considered non-pathogenic as there are no known PICV-associated diseases in humans or other animals [34]. The virus can infect a wide range of cell types from diverse host animal species that may include but are not necessarily limited to human, mouse, monkey, and avian species [34,35,36,37]. The wide host ranges and avirulent nature of PICV makes it a promising viral vector for flexible delivery of vaccine immunogens in humans and various animal species. 

A reverse genetics system has recently been developed to generate recombinant tri-segment PICV (rPICV) that carries as its genome two different small genomic (S) segments that encode the nucleoprotein (NP) and glycoprotein precursor complex (GPC), respectively, in each of the S segments and a long (L) segment that encodes the Z matrix protein and the L RNA-dependent RNA polymerase (RdRp). Additionally, the genome of the tri-segmented rPICV has been engineered to accommodate up to two foreign genes that can be used as vaccine antigens [34]. For instance, both HA and NP genes of a laboratory-adapted IAV strain PR8 have been simultaneously inserted into the rPICV genome to produce a rPICV vectored vaccine that express IAV HA and NP concomitantly. When administered into mice, this experimental vaccine induces high levels of HA-specific neutralizing antibodies and NP-specific T cell response and protects the mice against a lethal challenge with IAV PR8 infection [34]. Another rPICV vectored vaccine expressing turkey arthritis reovirus antigens has also been generated and shown to induce high serum neutralizing antibody titers compared with the nonimmunized turkey poults [36]. 

In the present study, a rPICV vectored vaccine expressing the HA antigen of an IAV-S H3N2 strain (designated rPICV-H3) was generated. High levels of the H3 antigen were expressed in different pig cell types that were infected with rPICV-H3. Pigs vaccinated with the rPICV-H3 vaccine developed high titers of neutralizing antibody and were protected against a challenge infection with the homologous IAV-S H3N2 strain. Collectively, this study provides a proof of concept that rPICV can successfully be used as a viral vector for anti-influenza vaccination. 

## 2. Materials and Methods

### 2.1. Cells, Reagents, and Viruses

Baby hamster kidney (BHK-21) and porcine kidney-15 (PK-15) cells were cultured in complete Dulbecco’s Modified Eagle Medium (DMEM) (Life Technologies, Grand Island, NY, USA), supplemented with 10% fetal bovine serum (FBS, Sigma-Aldrich, St. Louis, MO, USA) and 1× penicillin–streptomycin (100 units/mL of penicillin and 100 µg/mL of streptomycin, Life Technologies, Grand Island, NY, USA). Porcine alveolar macrophages (PAMs) were collected from lung lavage of pigs between 4 and 8 weeks old and were cryopreserved in a cell freezing medium containing 40% Roswell Park Memorial Institute (RPMI) (Life Technologies, Grand Island, NY, USA), 50% FBS, and 10% dimethyl sulfoxide. When needed, the cells were revived and cultured in complete RPMI supplemented with 10% FBS and 1× penicillin–streptomycin. Madin–Darby Canine Kidney (MDCK) cells were cultured in DMEM supplemented with 10% FBS, 1× penicillin–streptomycin, 0.2% Bovine serum albumin (BSA, Sigma-Aldrich, Saint Louis, MO, USA), and 25 mM HEPES (Hyclone, Life Technologies, Grand Island, NY, USA). All cell cultures were incubated at 37 °C in a humidified environment containing 5% CO2. 

The IAV-S strain A/swine/Texas/4199-2/1998 (H3N2 TX98) was obtained from The National Veterinary Services Laboratories (NVSL, Ames, IA, USA). The virus was propagated in MDCK cells in a virus inoculation medium (DMEM) supplemented with 1× penicillin–streptomycin, 0.2% BSA, 25 mM HEPES, and 1 µg/mL TPCK-treated trypsin (ThermoFisher Scientific, Rockford, IL, USA).

### 2.2. Experimental Vaccines

The recombinant HA protein of H3N2 TX98 (designated H3-protein) was expressed in a baculovirus expression system via a contract with Genscript (Piscataway, NJ, USA). The purified protein was emulsified in 20% (*v*/*v*) Emulsigen-DL 90 (Phibro Animal Health Corporation, Omaha, NE, USA). Each dose of this vaccine contained 100 µg protein in a total volume of 2 mL.

The rPICV expressing the HA antigen of H3N2 TX98 was constructed as previously described [34]. Briefly, the HA sequence was codon-optimized for optimal protein expression in pigs using a commercial DNA synthesis service (Genscript, Piscataway, NJ, USA). The gene was cloned into both S1 and S2 of the rPICV reverse genetics system [34,35]. The three plasmids containing S1, S2, and L segments were co-transfected into BSRT7 cells to rescue the rPICV expressing H3 antigen (designated rPICV-H3). The rPICV-H3 vaccine was amplified in BHK-21 cells, and viral titers were quantified by conventional plaque assay [34]. A rPICV expressing green fluorescence protein (rPICV-GFP) that was generated previously [34] was included in this study to serve as a vector control [34]. 

### 2.3. Antigen Expression In Vitro

BHK-21, PK-15, or PAMs cultured separately in 24-well plates were infected with rPICV-H3 and rPICV-GFP at a multiplicity of infection (MOI) of 1 pfu per cell. At 48 h post-infection (hpi), cells were washed once with PBS and fixed in 500 μL cold solution of methanol/acetone (1:1 *v*/*v*), followed by incubation with a monoclonal antibody specific to H3 antigen (generated in our laboratory) for 1 h at room temperature (RT). The cells were washed three times with phosphate buffer saline (PBS, pH 7.4) and incubated with a donkey anti-mouse Alexa flour 488 IgG H + L (Invitrogen, Life Technology corporation, Eugene, OR, USA) for 1 h at RT. After another three washes with PBS, fluorescent signals from cells were observed via an inverted fluorescence microscope. Fluorescent images were taken using the Nikon Eclipse Ts2R-FL operated by Nikon NIS Elements (ver 5.02). Cells infected with rPICV-GFP were directly visualized under fluorescence microscope 48 hpi. All images were taken using a 20× objective.

### 2.4. Animal Experiment

**Figure 1:**
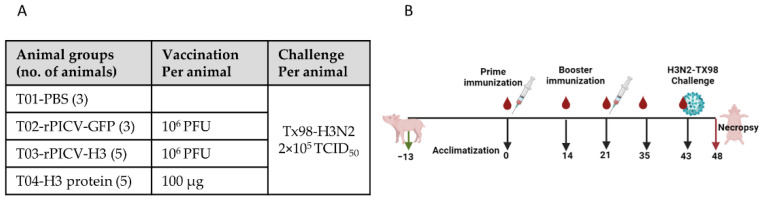
Animal study design (**A**) Table indicating different treatment groups that were included in the study along with dosage specification for vaccine and challenge virus. (**B**) schematic view of study schedule representing duration at which vaccination, challenge, and sample collections were carried out. Image was made using the Biorender tool.

Sixteen 4-week-old pigs seronegative for porcine reproductive and respiratory syndrome virus (PRRSV) and IAV-S were purchased from the University of Nebraska-Lincoln (UNL) research farm and were housed in the animal biosafety level 2 (ABSL2) research facility at UNL. Pigs were randomly assigned into 4 treatment groups (T01–T04). T01 had three pigs that were injected intramuscularly (IM) with 2 mL PBS to serve as non-vaccinated controls. T02 had 3 pigs that were injected IM with 10^6^ pfu of rPICV-GFP in 2 mL. Pigs in T01 and T02 groups were commingled in the same room throughout the course of the study. T03 contained five pigs that were vaccinated IM with 2 mL vaccine formulation containing 10^6^ pfu of rPICV-H3. T04 had five pigs that were vaccinated IM with 2 mL of the H3-protein subunit vaccine. The pigs were vaccinated twice on days 0 and 21 (Figure 1). One pig from T03 and another from T04 were humanely euthanized and removed from the study for welfare purposes due to injury during the blood collection process and for other reasons unrelated to rPICV infection, respectively.

Whole-blood samples with ethylenediaminetetraacetic acid (EDTA) anticoagulant were collected from all pigs at different time points before and after immunization and plasma samples were isolated to measure the humoral immune response. 

On day 43 post vaccination (pv), all pigs were challenged by an intra-tracheal inoculation with 2 × 10^5^ TCID_50_ of the H3N2 TX98. Nasal swabs were taken from all pigs daily post-challenge (pc) to measure potential IAV-S shedding. Pigs were humanely euthanized on day 48 pv (5 days post-challenge). Lung gross lesion was scored during necropsy by a veterinary pathologist who was blinded to the experimental treatment groups. Bronchioalveolar lavage fluid (BALF) samples were collected in cold PBS to measure viral genome copy number and infectivity. Samples of lung were fixed in 10% buffered formalin and processed by routine procedures for histopathologic examination as described in more details below. 

### 2.5. Serological Assay 

Antibody responses against IAV-S nucleoprotein (NP) were measured by the Iowa State University Diagnostic Laboratory using a commercially available blocking ELISA (IDEXX, Montpellier, France). Results are expressed as test sample to negative control optical density (OD) ratios (S/N ratio). Samples with S/N ratio below 0.6 was considered positive, as it indicated the presence of higher levels of anti-NP antibodies in the test sample than in the negative control used in the assay. 

Antibody responses against the H3 protein and the GFP were measured using ELISA. Briefly, flat-bottom 96-well plates were coated overnight at 4 °C with 100 µL of either the H3 protein or the GFP diluted in PBS to the final concentration of 2 µg/mL or 5 µg/mL, respectively. After washing with PBS containing 0.1% tween 20 (PBS-T20), the wells were blocked with 250 µL/well of a blocking buffer (10% skim milk in PBS-T20) for 2 h at RT. Plasma samples were diluted in an assay buffer (5% skim milk in PBS-T20), and 100 µL of diluted sample was added to each well of the plates in duplicate, followed by 1 h incubation at RT. After three washes with PBS-T20, 100 µL of HRP-labeled goat anti-pig IgG (Sera Care, Milford, MA, USA) diluted 1:5000 in assay buffer was added to each well followed by 30 min incubation at room temperature. After three washes with PBS-T20, 100 µL of ABTS substrate (Sera Care, Milford, MA, USA) was added to each well. The plate was incubated at RT between 5 and 25 min (until color was observed in the positive control wells). The reaction was stopped by adding 100 µL of 1% sodium dodecyl sulfate (1% SDS) diluted in distilled water. Optical density (OD) was measured at 405 nm wavelength by an ELISA plate reader (Bio-Tek ELx808). For the H3 ELISA, samples were diluted 2-fold serially before adding to the ELISA plates. An arbitrary cutoff value equivalent to mean plus five standard deviations of the OD values of plasma samples from the non-immunized control animals was calculated. Antibody titers were determined at the highest dilutions with OD values above the cutoff value. For GFP- ELISA, plasma samples were tested at the dilution 1:100 and data are presented as the OD_405_ value of each sample at the 1:100 dilution. 

Virus neutralization assay and hemagglutination inhibition assay were essentially performed as previously described [38].

### 2.6. Quantification of Viral Load

RNA was extracted from nasal swabs and BALF samples using Quick RNA viral Kit (Zymo Research, Costa Mesa, CA, USA) according to the manufacturer’s protocol. Viral genomic copies were quantified using a real-time reverse transcription PCR (RT-PCR) by VetMax-Gold SIV Detection Kit (Life Technologies, Austin, TX, USA), as previously described [38]. Chemically synthesized RNA fragment with known copy numbers was used to establish a standard curve based on which the absolute copy numbers of viral RNA in each sample was estimated. Viral loads in nasal swabs were reported as log_10_ copies per μL of RNA used for the RT–PCR reaction. For statistical purposes, samples with undetectable levels of viral RNA were assigned a zero value.

Infectious IAV-S titers in BALF samples were determined by virus titration assay in MDCK cells cultured in 96-well plates. Briefly, the samples were diluted 10-fold serially in the virus inoculation medium, and 100 µL of each dilution was inoculated in each well for a total of four wells per dilution. An immunofluorescence assay (IFA) was performed at 48 h to visualize the infected cells, and virus titer was calculated following the Reed–Muench method for estimating fifty percent endpoint [39].

### 2.7. Lung Pathological Analysis

Lung gross lesions were evaluated during necropsy by a veterinary pathologist who was blinded to the treatment groups. The percentage of lung consolidation was calculated as described previously [40]. Sections of three different lung lobes, cranial, middle, and caudal, were stained with hematoxylin and eosin (H&E) following a routine pathological procedure. The slides were evaluated by a veterinary pathologist who was blinded to treatment groups following the scoring parameters as described previously [22]. Each of the three lung lobes was scored for six different parameters to generate a composite score ranging from 0 to 22 as previously described [22].

RNA in situ hybridization (ISH) was performed in the lung sections as previously described [38]. Composite scoring for the ISH slides was performed similarly to the composite score for microscopic lesions.

### 2.8. Statistical Analysis

All statistical analyses were carried out using the GraphPad Prism 9.0. Antibody titers were log_2_ transformed and analyzed using the mixed-effects analysis multiple comparisons. Gross lung lesion score, lung microscopic lesion score, viral genome copies, and virus titers were analyzed by ordinary one-way analysis of variance (ANOVA), followed by Tukey’s multiple comparison test. 

## 3. Results

### 3.1. rPICV Vector Expressed High Levels of Gene-of-Insterest in Pig Cells

**Figure 2:**
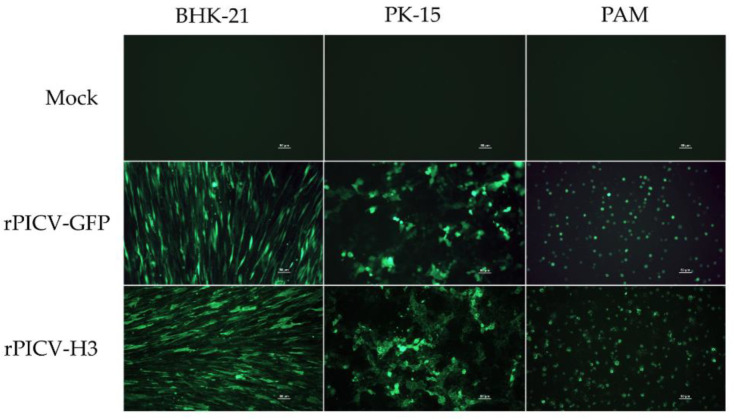
Expression of genes-of-interest. BHK-21, PK-15, and PAMs were infected with either rPICV-GFP or rPICV-H3 viruses at an MOI of 1 pfu per cell. At 48 hpi, GFP images were taken directly from cells infected with the rPICV-GFP virus. On the other hand, IFA was performed to detect expression of HA protein in cells infected with the rPICV-H3 virus. Bar = 100 μM.

We first evaluated the expression of the gene-of-interest (either HA antigen or GFP) in two types of pig cells: PK-15 and PAMs. BHK-21, which is routinely used to propagate rPICV, was included in the study for comparative purposes. The cells were infected with either rPICV-H3 or rPICV-GFP at an MOI of 1 pfu per well of the 24-well plate. At 48 hpi, GFP expression was observed in all three types of cells: BHK-21, PK-15, and PAM (Figure 2). An IFA was performed to detect HA antigen in cells infected with rPICV-H3 virus. HA-positive cells were observed in all three cell types tested. Collectively, the results indicate that rPICV can deliver genes-of-interest into pig cells.

### 3.2. Pigs Inoculated with rPICV-GFP Do Not Transmit the Virus to Contact Pigs 

**Figure 3:**
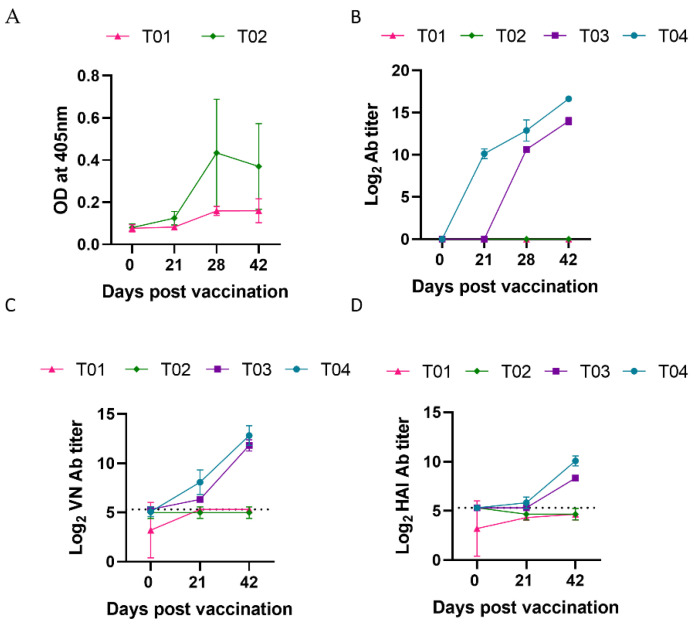
Humoral antibody responses post vaccination. (**A**) Anti-GFP antibodies in plasma samples from the T01 (PBS) and T02 (rPICV-GFP) groups. (**B**) Anti-H3 antibodies in plasma samples from all treatment groups. An arbitrary cutoff equivalent to mean plus 5 standard deviations of OD values of samples from the T01 and T02 groups was calculated. Samples with OD values greater than the cutoff were considered to be positive. The end-point antibody titers were expressed as the log_2_ of the reciprocal of the highest plasma dilution that tested positive by the H3-specific ELISA. (**C**) Virus neutralization (VN) antibody titers against H3N2 TX98. (**D**) Hemagglutination inhibition antibody titers against H3N2 TX98. In (**C**,**D**), the horizontal dotted lines at 5.32 log2 (1:40 dilution) indicate the background antibody titers found in all pigs prior to vaccination. T01—PBS; T02—rPICV-GFP; T03—rPICV-H3; T04—H3-protein.

To determine whether rPICV can spread horizontally from one pig to another, the pigs that were injected with rPICV-GFP (T02) were commingled with the PBS-injected group (T01) throughout the course of the study. Antibodies against GFP were measured in plasma samples collected at multiple time points before and after vaccination using an indirect ELISA. Anti-GFP antibodies were detected in the T02 group starting from day 28 pv but not in group T01 (Figure 3A). The result suggests that pigs vaccinated with rPICV-GFP did not transmit the virus to induce an anti-GFP antibody response in the contact pigs. 

### 3.3. Pigs Immunized with rPICV-H3 or H3-Protein Subunit Vaccines Mounted a Robust Antibody Response 

Antibodies specific to the H3 antigen were measured by an indirect ELISA. As expected, H3-specific antibodies were only detected in T03 and T04, not in the T01 and T02 groups (Figure 3B). H3-specific ELISA antibodies were detected in T04 pigs starting from day 14 pv and continued to increase on days 28 and 35 pv. H3-specific ELISA antibodies were not detected in T03 pigs until day 28 pv, corresponding to day 7 post-boost. The results indicate that the H3-protein subunit vaccine elicited faster antibody responses than the rPICV-H3 vectored vaccine. However, no significant difference in the H3-specific ELISA antibody titers were observed between T03 and T04 after day 28 pv (corresponding to day 7 post-boost).

Next, virus neutralization (VN) and hemagglutination inhibition (HI) antibody titers were evaluated against H3N2 TX98, the homologous strain from which the HA gene was derived to generate the H3-protein and rPICV-H3 viral vectored vaccine used in this study. Background levels of VN and HI antibody titers (approximately 1:40) were observed in all pigs before vaccination and were maintained at similar titers in the T01 and T02 groups throughout the study (Figure 3C,D). In contrast, VN and HI antibody titers significantly increased in the T03 and T04 groups, starting from day 21 pv, and further increased at day 42 pv. VN and HI antibody titers were not significantly different between the T03 and T04 groups at day 42 pv (Figure 3C,D). The results indicate that both the rPICV-H3 vectored and H3- protein vaccines were able to elicit neutralizing antibodies in pigs.

### 3.4. Pigs Immunized with rPICV-H3 and H3-Protein Vaccines Were Protected against Challenge with the Homologous H3N2 IAV-S Strain 

**Figure 4:**
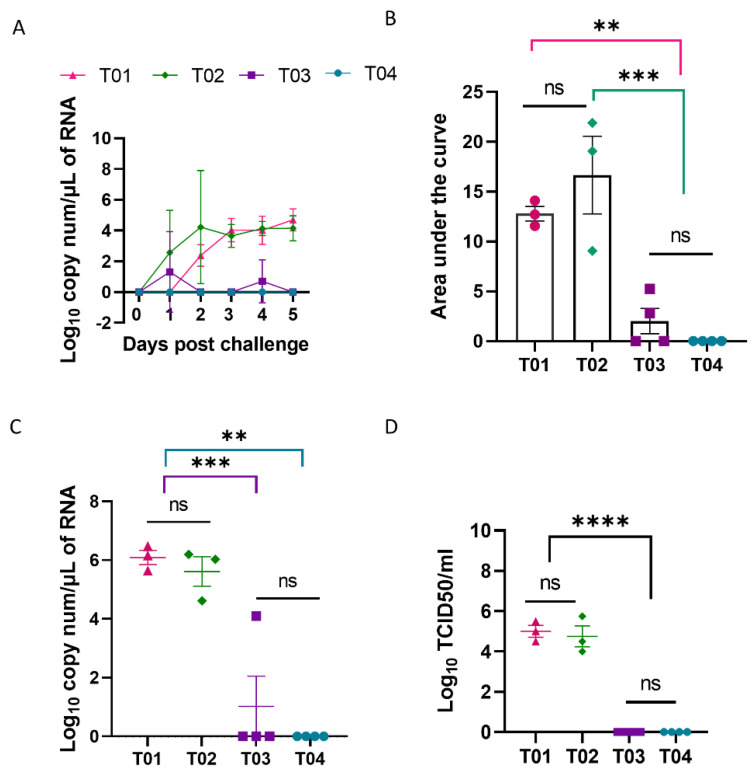
IAV-S shedding post challenge. (**A**) IAV-S NP RNA in nasal swab samples as quantified by RT-PCR. Data are expressed as log_10_ RNA copy per µL of RNA sample loaded to the PCR reaction. (**B**) Area under each curve of the nasal viral loads in pigs in the course of 5 days post challenge infection with IAV-S. (**C**) IAV-S NP RNA in BALF collected during necropsy at day 5 post challenge. (**D**) Infectious IAV-S in BALF samples measured by virus titration in MDCK cells. T01—PBS; T02—rPICV-GFP; T03—rPICV-H3; T04—H3-protein. ns—no significance, ** *p* ≤ 0.01, *** *p* ≤ 0.001, **** *p* ≤ 0.0001.

All pigs were challenged by an intra-tracheal inoculation with the H3N2 TX98 strain at day 43 pv (corresponding to day 22 post-boost). Nasal swabs were collected daily to measure IAV-S shedding. Viral RNA was detected in nasal swabs of all pigs in the T01 and T02 groups, starting from day 1 pc. In contrast, IAV-S RNA was not detected in any of the pigs in the T04 group at any sampling days (Figure 4A). For group T03, two pigs had detectable levels of IAV-S RNA at one sampling day while the remaining two pigs did not have any detectable levels of viral RNA at any sampling days. To compare the total levels of IAV-S shedding among treatment groups, area under the curve (AUC) was calculated for individual pigs for the course of 5 days post challenge with IAV-S. The AUC of the T03 group was similar as that of the T04 group and was significantly lower than that of T01 and T02 (Figure 4B). 

All pigs were humanely sacrificed at day 5 post challenge, and a BALF sample was collected to measure IAV-S RNA by using an RT-PCR. High levels of IAV-S RNA were detected in the BALF from all pigs in T01 and T02 (Figure 4C). In contrast, no IAV-S RNA was detected in the BALF from any of the pigs in group T04. One out of four pigs in the T03 group had a detectable level of IAV-S RNA in the BALF. This pig also had IAV-S RNA in its nasal swab collected on day 4 pc. To determine IAV-S infectivity, samples of BALF were titrated on MDCK cells. All BALF samples collected from T01 and T02 had infectious IAV-S with the titers ranging from 10^4.0^ to 10^5.75^ TCID_50_/mL. In contrast, infectious IAV-S was not detected in the BALF of any pigs in T03 and T04, even though one pig in the T03 group had a detectable level of IAV-S RNA as determined by RT-PCR (Figure 4D). 

**Figure 5:**
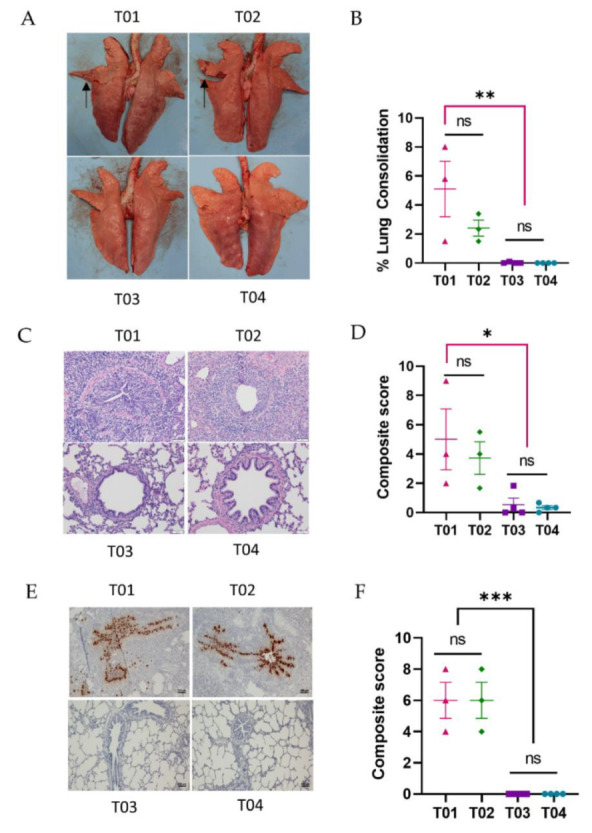
Lung macroscopic and microscopic lesion. (**A**) Representative pictures of pig lungs taken during necropsy. Black arrows indicate lung areas with typical consolidation caused by IAV-S infection. (**B**) Percentage consolidation calculated on a weighted average of each lung lobe. (**C**) Representative images of lung sections stained with H&E to evaluate microscopic lesions. (**D**) Composite microscopic lesion scores based on parameters described previously [22]. Sections from three different lung lobes (apical, middle, and caudal) of each pig were scored, and the average composite score of each pig was used for statistical analysis. (**E**) Representative images of ISH staining for IAV-S NP RNA in lung sections. (**F**) Composite ISH scores based on parameters described in [22]. T01—PBS; T02—rPICV-GFP; T03—rPICV-H3; T04—H3-protein. ns—no significance; * *p* ≤ 0.05; ** *p* ≤ 0.01; *** *p* ≤ 0.001.

At necropsy, gross lung lesions were evaluated. Mild lung consolidation was observed in all pigs in the T01 and T02 groups, with the percentage of consolidated lung surface varying from 1.5% to 8%. Pigs in the T03 and T04 groups did not exhibit any visible lung consolidation (Figure 5A). 

During necropsy, samples from three lung lobes (apical, middle, and caudal) were selected for microscopic evaluation. Average composite scores of the three lobes are reported (Figure 5C). Lung sections from the T01 and T02 groups exhibited mild-to-moderate interstitial pneumonia with necrotizing bronchiolitis and peribronchiolar lymphocytic cuffing (Figure 5C). The mean composite microscopic scores of the T01 and T02 groups ranged from 1.7 to 9. On the contrary, no significant microscopic lesions were observed in the lung sections of pigs in the T03 and T04 groups (Figure 5D). 

In situ hybridization (ISH) was used to detect IAV-S NP gene transcript in the cardiac (middle) lung lobe of all pigs (Figure 5E). NP transcripts were detected in all lung sections of pigs in the T01 and T02 groups. Conversely, no viral NP transcripts were observed in any lung section of pigs in the T03 and T04 groups (Figure 5F). 

Collectively, the results demonstrate that both the rPICV-H3 or H3-protein vaccines protected pigs from the challenge infection with a homologous H3N2 strain.

### 3.5. Pigs Immunized with H3-Protein Vaccine or rPICV-H3 Vectored Vaccine Tested Negative for IAV-S NP Antibodies by a Commercial ELISA Kit

**Figure 6:**
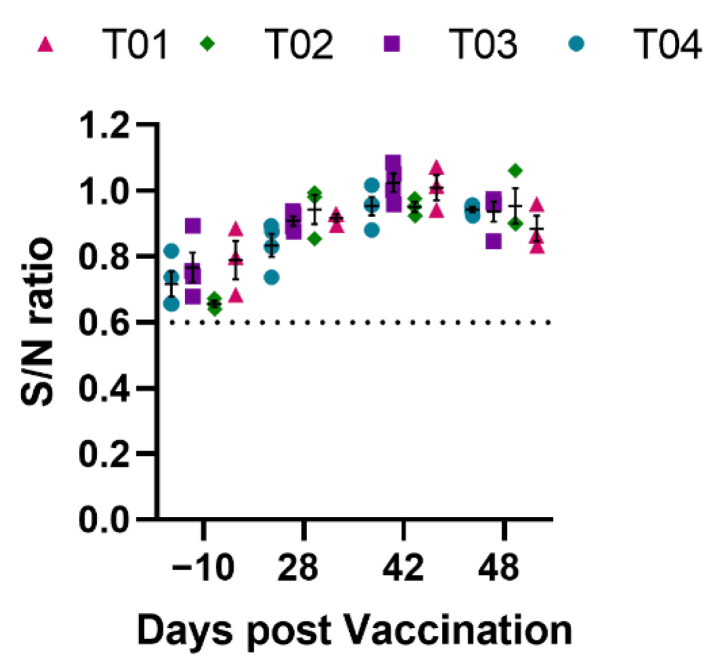
Antibodies against IAV-S NP protein measured by a blocking ELISA. Data are expressed as the sample to negative (S/N) ratio. The horizontal dotted line at S/N of 0.6 is the assay cutoff. Test samples with S/N greater than 0.6 are considered to be negative. T01—PBS; T02—rPICV-GFP; T03—rPICV-H3; T04—H3-protein.

Antibodies specific to IAV-S NP were measured using a commercial ELISA kit that is routinely used for IAV-S serodiagnosis. All pigs tested negative for IAV-S NP antibody before vaccination (day -10) and continued to be negative till the end of the study (day 48 pv). The results demonstrate that the experimental vaccines used in this study did not induce antibody against IAV-S NP protein. Thus, vaccination with H-3 protein or rPICV-H3 vectored vaccine does not interfere with the serodiagnosis of IAV-S (Figure 6). 

## 4. Discussion

The HA is a viral envelope protein responsible for virus attachment to the cellular receptor [41,42]. Consequently, it is the main target for the development of vaccines against influenza virus [43]. Immunization of pigs with a purified HA protein expressed in a mammalian expression system resulted in complete protection against homologous virus challenge [25]. However, pigs vaccinated with HA-protein subunit vaccine exhibited VAERD when they were challenged with an antigenically mismatched strain [44]. On the other hand, vaccination with a replication-defective Ad5 viral vector expressing HA did not result in VAERD upon challenge with an antigenically mismatched IAV-S strain [45]. These data suggest using a viral vector to deliver HA antigen is an attractive approach for the development of IAV-S vaccines. 

The main objective of this study was to evaluate the immunogenicity and protective efficacy of an rPICV viral vector expressing HA antigen of the IAV-S H3N2 strain in pigs (rPICV-H3). PICV is a non-pathogenic virus that was originally isolated from rats [33]. The viral genome has been engineered to allow it to carry up to two different vaccine immunogens. It has been demonstrated previously that rPICV viral vector effectively delivers vaccine immunogens in mice and turkeys [34,36]. In the present study, we demonstrated that pigs immunized with the rPICV-H3 vector vaccine mounted a robust humoral immune response and were completely protected against challenge infection with the homologous IAV-S H3N2 strain.

Pathogenesis of PICV infection in pigs has not been studied yet. There is no information pertaining to the target tissue and cell types for PICV replication, routes, and duration of viral shedding. Hence, we were interested in determining whether the pigs infected with rPICV spread the virus. For this purpose, pigs vaccinated with rPICV-GFP (T02) were commingled with those injected with PBS (T01). The rationale is that if pigs vaccinated with rPICV-GFP shed the virus, the naïve contact pigs (injected with PBS) would develop antibodies against GFP. Anti-GFP antibodies were detected in pigs vaccinated with rPICV-GFP but not in the contact pigs. Thus, our preliminary data suggest that pigs vaccinated with rPICV do not spread the pichinde virus horizontally to the contact pigs. 

It has been well-documented that vaccination of pigs with an HA-protein-based vaccine will result in complete protection [25]. Therefore, we included a group of pigs that were immunized with a purified H3-protein vaccine (T04) in this study for comparative purposes. As expected, pigs vaccinated with the H3-protein vaccine developed a robust humoral antibody response, which can be detected at day 21 pv and continued to increase post boost. On the contrary, H3-specific antibodies were only detected at day 28 pv, corresponding to day 7 post boost in pigs vaccinated with rPICV-H3 vectored vaccine (T03). Although pigs in the T03 group developed antibody responses later than those in the T04 group, their VN- and HAI-antibody titers sharply increased and reached similar titers compared to those in the T04 group by day 42 pv. In previous studies, the amount of protein mass used to vaccinate pigs ranged from 0.5 μg to 25 μg per pig per immunization, which were sufficient to confer sterilizing immunity against the challenge infection with a homologous H1N1 strain [25,26,44]. In this study, we immunized pigs with 100 μg purified H3 protein, which was significantly higher than the antigenic dose used in previous studies [44]. We believe that the earlier antibody response observed in pigs immunized with the H3-protein vaccine was partially due to the higher antigenic mass used to vaccinate pigs. 

None of the pre-vaccination samples had antibodies against IAV-S NP as determined by a commercial ELISA (Figure 6). Similarly, none of the pre-vaccination samples had antibodies against HA protein of H3N2 TX98 (Figure 3B). However, samples collected prior to the study exhibited some level of HAI- and VN-antibody titers against H3N2 TX98 challenge strain (titer ≤ 1:40, equivalent to 5.32 Log_2_). VN- and HAI- titers maintained the same or slightly declined levels by day 42 pv in pigs injected with PBS (T01) or with rPICV-GFP (T02). In contrast, these antibody titers rapidly increased in pigs vaccinated with the H3-protein subunit vaccine (T04) or rPICV-H3 vectored vaccine (T03). Thus, the VN- and HAI-antibody titer in samples collected pre-study might be due to non-specific and/or cross-reactivity. It was reported recently that antisera from SARS-CoV-2 patients could cross-react with the IAV HA protein [46]. The pigs used in this study were unlikely to be infected with SARS-CoV-2. Instead, they might be pre-exposed to one of the porcine coronaviruses, which are widely prevalent in swine herds [47]. 

After challenge infection, viral RNA was not detected in nasal swabs, BALF, and lung section of pigs in the T04 group. Thus, under the experimental conditions of this study, the H3-protein vaccine conferred sterilizing immunity against the homologous H3N2 strain. Two pigs in T03 exhibited detectable levels of IAV-S RNA in their nasal swabs at one sampling day and one of these two pigs had detectable levels of viral RNA in its BALF sample at day 5 post challenge. When titrated in MCDK cells, the BALF sample in the T03 pig that contained IAV-S RNA, as determined by RT-PCR, did not show evidence of successful IAV-S infection, indicating that the BALF sample was non-infectious or at levels below the limit of detection of the assay. Statistically, the viral IAV-S RNA copies in nasal swabs and BALF were not significantly different between the T03 and T04 groups. Likewise, the lung lesion scores were not different between the T03 and T04 groups, both in terms of gross- and microscopic-lesion. Collectively, the results suggest that the rPICV-H3 vectored vaccine confers an equivalent level of protection as compared to the H3-protein vaccine. 

ELISA kits detecting antibodies specific to the NP are available for the serodiagnosis of IAV-S. All pigs in the T03 and T04 groups tested negative by the NP ELISA kit throughout the course of the study, even though they exhibited high antibody titers against the HA protein. Thus, pigs vaccinated with the H3-protein vaccine or with the rPICV-H3 vectored vaccine can be serologically distinguished from those that are naturally infected with IAV-S by NP ELISA. Therefore, the H3-protein subunit and rPICV-H3 vectored vaccine used in this study fulfills the requirement of a DIVA (differentiating infected from vaccinated animals) vaccine. It should be noted that antibodies against the IAV-S NP protein were not detected in pigs in the T01 and T02 groups at day 5 post challenge infection (corresponding to day 48 pv). We believed that this was too soon after infection for pigs in the T01 and T02 groups to mount an antibody response to the NP protein. Indeed, anti-NP antibodies were only detected in a few pigs that were experimentally inoculated with H1 and H3 IAV-S at day 14 post infection [48] 

## 5. Conclusions

rPICV is a promising viral vector for the development of IAV-S vaccines. Our results demonstrate that the rPICV vectored vaccine does not spread horizontally among pigs. Importantly, pigs vaccinated with the rPICV-H3 vectored vaccine induced HAI and VN antibodies and were fully protected against challenge infection with a homologous IAV-S strain. Additional studies are needed to determine whether pigs vaccinated with a rPICV-vector expressing HA antigen followed by an infection with a mismatched IAV-S strain can induce a cross-protective antibody or develop VAERD.

## Data Availability

Not applicable.
